# Practical Notes and Formulæ

**Published:** 1881-04-20

**Authors:** 


### PRACTICAL NOTES AND FORMULÆ.

**Raw Meat Juice in Cases of Inanition from Irritable Stomach.**—Dr. Hendree, of Alabama, writes: "A month since I was called by a school teacher, living eight miles distant, to visit his child eighteen months old, which was in a deplorable state of emaciation from a diarrhœa of three weeks duration. This was, in a measure, checked, but the irritation had extended to the stomach, and no nourishment, not even a teaspoonful of water could be retained. Enemata of beef tea and milk had been unsuccessful, and the father stated that unless soon relieved his child would starve to death.

An impending obstetrical case prevented me from leaving home, and knowing that Valentine's raw meat juice had been successfully used in a number of hospitals, I gave him a bottle with explicit directions, promising to go next day if desired.

Heard nothing farther, until a month later I was called by Mr. Young to his little girl of three years, dreadfully burnt by her clothes taking fire in the absence of the mother, the previous day. The father stated in a note that the child called constantly for drink and nourishment but could retain nothing whatever.

"I found an excoriating burn from the knees to the chin, including thighs, genitals, abdomen, entire front of throat, arms, hands, not neck and chin; all denuded of cuticle; the pulse indicated much depression and, after soothing dressing I left a bottle of meat juice which was retained and relished from the first.

At a second visit, some days later, I found the child recovering, and as the family of the patient first alluded to lived on the next farm, I called on my way home. The mother brought the child out perfectly well, running about, and stated that the meat essence was the first thing retained; had all been used, and she believed had saved the child's life."

**For the Anemia of Chlorosis.**—

℞ Ferri vini amari .................................... ℥ vijss.

Tinct. nucis vomicæ .................................... ʒ ij.

Liq. potass. arsenit .................................... ʒ ij.

M. Sig. A dessertspoonful in a glassful of water just after each meal.—*Prof. T. Gaillard Thomas, M. D.*

In addition to this Dr. Thomas (regarding the indications as to remove the cause, cure the neurosis and repair the damages), advises general tonic treatment and the observance of good hygiene.—*Medical Gazette.*

**Simple Continued Fever.**

℞ Acid. hydrobrom .................................... ʒ j.

Syr. simplicis .................................... ʒ ij.

Aq .................................... ℥ j.

M. Sig. Every hour.—*Fothergill.*

Dr. Fothergill, in speaking of the above formula, says it will probably constitute *par excellence* the fever mixture of the future. It is especially indicated where there is cerebral disturbance.—*Ibid.*

**Sore Nipples.**—

℞ Aquæ rosæ,

Glycerine.............................................. aa ℥ ij.

Acidi tannici.............................................. ʒ ij.

Ft. lotion.

Sig. Soak lint in this solution and apply to nipples.—*Dr. Baker.*

If the ulcerative process has commenced, it is advisable to stop nursing and paint the nipple with a solution of nirate of silver, 10 grs. to the ounce of distilled water.—*Ibid.*

**In the Pruritus of Pregnancy.**—

℞ Thymol.............................................. gr. xv.
Vaseline.............................................. gr. xxx.
Powdered brick clay.............................................. ℥ iij.

Dissolve the thymol in the vaseline and rub it up with the clay.—*Professor Montrose A. Pallen, M. D.*

This is to be applied to the pruritic parts, washed off every day or two and re-applied.

Dr. Pallen's experience has been, that excepting those cases depending on trophic nervic causes, this prescription will always effect a cure. He advises its use also in herpes and similar eruptions accompanying later months of gestation.—*Ibid.*

**Cystitis.**—

℞ Acidi benzoici,
Sodii biboratis,.............................................. aa gr. x.
Inf. buchu.............................................. ℥ ij.

This amount three or four times a day.—*A. F. C. Skene, M. D.*

This may almost be called a specific in its influence in the earlier stages of cystitis, affording rapid and lasting relief. The diet should be carefully regulated, and the skin and bowels kept in active condition.—*Ibid.*

**Remedy for Frost-Bite.**—Nordenskjold used upon his Arctic expedition an ointment prepared as follows:

Corrosive Sublimate.............................................. 1 gramme.
Castor Oil..............................................40 drops.
Oil Turpentine..............................................60 drops.
Collodion..............................................40 grammes..

—*Pharm. Zeitung fur Russland.*

**Antidote for Carbolic Acid.**—Sulphuric acid in moderate doses is recommended by Dr. Stanfleben, of Russia, as an antidote for carbolic acid The two acids are said to combine, and to form a non-poisonous compound. According to the Medical Herald, of Louisville, Ky., it is to be given in simple syrup and the syrup of gum-arabic. Success depends upon its early administration.—*Chicago Medical Review.*

**Remedies for Spermatorrhœa.**—Tincture of gelseminum (green-root) in doses of thirty drops, 3 times a day, has an excellent effect in checking nocturnal emissions.

℞ Sulphate zinc,

Pulv. rhubarb,

Extract hyoscyamus, aa 2 grains,

Extract belladonna, ¼ grain.

For one pill. To be taken three times a day until the effects of the belladonna are noted, then twice daily, with:

℞ Bromide ammonium.................................... ℥ ss

Tinct. lupulin.......................................... ℥ j

Camphor water........................................ ℥ iij.

Mix. Tablespoonful at bedtime.

Helonias dioica has proved successful in doses of 10 to 15 grains of the crude root, pulverized, 3 times a day.

Dr. Adolphus considers senecio gracilis one of the best remedies, in dose of half teaspoonful, powdered, in water, 3 times a day. Also speaks well of cannabis indica resin, dose, half a grain three times a day.

A writer in one of the English Journals states that nocturnal emissions occurring in healthy young men not addicted to self-abuse, may be entirely kept in check by drachm doses of tincture sesquichloride of iron.

The following is an old and, in many cases successful formula:

℞ Gelsemin, 8 grains,
Lupulin, 48 grains.

Mix and divide in 16 powders. Dose one at bedtime.

Aconitine 1-16 grain at bedtime, is said to have cured obstinate cases.

Dr. Mitchell used ergot satisfactorily, giving it in doses of one-half drachm to a drachm daily, of the freshly powdered drug.

In most cases, owing to the relaxed condition of the mouth of the ejaculatory ducts, injections of astringents, as alum, hamamelis, etc., will be of benefit. A suspensory bandage should also be worn, and the following rules observed:

1. Avoid all sources of sexual excitement.
2. Bathe the parts with cold water, 10 minutes night and morning.
3. Evacuate the urine before going to bed.
4. Sleep on a hard bed with light covering, lying on the right side.
5. Arise at the first awakening in the morning.
6. Avoid the use of the stimulants, tea, coffee, tobacco, and medicines or drinks of a diuretic nature.—*New York Medical and Surgical Journal.*

**Remedy for Corns.**—Mr. Gezow, a Rusian apothecary, recommends tbe following as a "sure" remedy for corns, stating that it proves effective in a short time, and without causing any pain: Salicylic acid, 30 parts; extract of cannabis indica, 5 parts; collodion, 240 parts. To be applied by means of a camel's hair pencil.—*Phar. Zeit.*

**Ipecac in Dyspepsia.**—Dr. Fothergill says : Ipecacuanha formed a portion of a good old-fashioned dinner pill; and betwixt its direct action upon the gastric mucous membrane, and its action upon the liver as an hepatic stimulant, it must come into use again before long. A dinner pill of—

| | |
|---|---|
| Pul. ipecacuanhæ | gr. j. |
| Strychniæ | gr. 1-40. |
| Pulv. pip. nig | gr. ij |
| Pil. aloe et myrrh | gr. ijss. |

Every day will often produce excellent effects. Then arsenic may be taken as three drops of Fowler's solution after dinner; or in the above pill substituting the same dose of arsenic for the strychnine.

**Copaiva in Sciatica.**—Dr. H. C. Marsh writes to the London Medeical Time's and Gazette, of copaiva:

I wish to speak of this drug as wonderfully efficacious in sciatica. The treatment of sciatica seems to be a little indefinite. I fancy we all meet with very troublesome instances in which we administer a variety of drugs with a perseverance that deserves, but does not always command success. (After describing a most obstinate case he stated) at last I prescribed

| | | |
|---|---|---|
| ℞ | Bals. copaib | ʒ iv. |
| | Tr. lavand | ʒ iv. |
| | Tr. hyos | ʒ iij. |
| | Pot. bicarb | ʒ j. |
| | Mucilag | ℥ j. |
| | Aquæ | ℥ vj. M. |

A tablespoonful every four hours.—*Med. and Surg. Reporter.*

**Gleet.**—Having hit upon a remedy with which I am having good success in the treatment of this disease, I deem it my duty to place it before the readers of your valuable journal. It is the following :

℞ Zinci. Sulph. 12 grains,
Tinct. Iodine, 10 drops,
Water 8 ounces.

M. Sig. Inject four times a day.

Also—

℞ Ext. Uva Ursi fld, 3 ounces,
Ext. Pareira brav. 1 ounce,
Ext. Cascara sag. 2 ounces,
Syr. Orange peel, 2 ounces,
Water q. s. ad 8 ounces.

M. Sig. Teaspoonful three times a day before meals.

I consider this as a valuable remedy in obstinate cases.—*S. L. Blake, M. D., in Medical Brief.*

